# Shen Shuai II Recipe Attenuates Apoptosis in 5/6 Renal Ablation/Infarction Rats by Inhibiting p53 and the Mitochondrial Pathway of Apoptosis

**DOI:** 10.1155/2020/7083575

**Published:** 2020-02-07

**Authors:** Meng Wang, Jing Yang, Chen Wang

**Affiliations:** ^1^Department of Nephrology, Shuguang Hospital Affiliated to Shanghai University of Traditional Chinese Medicine, Shanghai 201203, China; ^2^Key Laboratory of Liver and Kidney Diseases, Ministry of Education, Shanghai University of Traditional Chinese Medicine, Shanghai 201203, China; ^3^TCM Institute of Kidney Disease, Shanghai University of Traditional Chinese Medicine, Shanghai 201203, China; ^4^Shanghai Key Laboratory of Traditional Chinese Clinical Medicine, Shanghai University of Traditional Chinese Medicine, Shanghai 201203, China

## Abstract

*Background*. Chronic kidney disease (CKD) is a global health burden with high mortality and morbidity. Clinical efficacy has been demonstrated for Shen Shuai II Recipe (SSR), an approved and widely used Chinese herbal medicine for over 20 years in China, to attenuate CKD progression. In this study, we explored the underlying molecular mechanisms of SSR benefits and studied its effects on apoptosis, a critical process in CKD development and progression. CKD was induced in rats with 5/6 renal ablation and infarction (A/I). Eight weeks after SSR treatment, we mainly assessed the severity of renal injury and fibrosis, the translocation of apoptotic factors in the mitochondrial apoptosis pathway, the degree of mitochondrial dysfunction, and the nuclear and mitochondrial translocation of p53. Furthermore, we detected the interaction of p53 with antiapoptotic Bcl-xL and Bcl-2 proteins. Our results showed that SSR significantly attenuated renal injury and fibrosis and inhibited the mitochondrial accumulation of proapoptotic proteins Bax and Puma and release of cytochrome c from mitochondria to the cytosol in a rat CKD model. In addition, SSR also improved the mitochondrial function and inhibited the nuclear and mitochondrial translocation of p53. In addition, SSR suppressed the p53 transactivation and the interaction of p53 with Bcl-xL and Bcl-2. These results suggested that SSR could block apoptosis in CKD by inhibiting p53 transcriptional-dependent and transcriptional-independent proapoptotic function and the mitochondrial pathway of apoptosis.

## 1. Introduction

Chronic kidney disease (CKD) has considerably increased worldwide and remains a major global health problem [1]. Different types of CKD ultimately progress to the end-stage renal disease (ESRD) through the same pathological process of renal fibrosis, resulting in a decline in quality of life and a substantial economic burden [2]. Therefore, finding cost-effective treatment of CKD is a key challenge for public health.

Apoptosis is a cell self-destruction process involved in a variety of biological events [3]. Abnormal apoptosis is implicated in various diseases related to ischemia, autoimmunity, and viral infection and contributes to pathological progression in the course of acute and chronic renal injury [4, 5]. In mammalian cells, the mitochondrial pathway of apoptosis, the so-called intrinsic pathway, plays a crucial role in activating apoptosis. Mitochondria are not only the site where Bcl-2 family members interact but also the origin of signals that initiate the activation of caspases through the release of apoptotic factors, such as cytochrome c [3]. P53, previously described as a tumor suppressor, is now considered to be a key component of the mitochondrial apoptosis pathway under cell stress. In response to various stimuli, such as hypoxia, DNA damage, and reactive oxygen species (ROS), p53 induces cell death through dual action, including activation of target genes and a transcriptional-independent signal mechanism [6].

It has been demonstrated that Chinese herbal medicine may be suitable for the treatment of CKD for its efficacy and safety of long-term treatment. Indeed, Shen Shuai II Recipe (SSR), a classic formula for the treatment of CKD in the clinic, has been widely used for more than 20 years and is an effective treatment option for CKD. Our previous studies have demonstrated that SSR attenuated renal fibrosis, increased renal blood flow, and improved oxygen consumption after renal injury in CKD induced by 5/6 renal ablation/infarction (A/I) in rats [7–9]. In this study, we aimed to elucidate whether SSR could exert antiapoptotic effects by P53 and the mitochondrial pathway of apoptosis.

## 2. Materials and Methods

### 2.1. Animals and Drugs

Eight-week-old male Sprague-Dawley (SD) rats, weighing approximately 190 g-210 g, were purchased from SLAC Laboratory Animal Co., Ltd. (Shanghai, China). Rats were maintained at 23 ± 3°C and relative humidity of 55 ± 15% with a 12 h light/12 h dark cycle and had free access to food and water. SSR consists of Salvia miltiorrhiza Bge (15 g), Codonopsis pilosula (15 g), Angelica sinensis (15 g), Rheum palmatum (15 g), Folium perillae (15 g), Epimedium (15 g), peach kernel (15 g), Ligusticum chuanxiong (15 g), and Coptis chinensis (6 g). The raw herbs for preparation of SSR were obtained from Shanghai Kangqiao Chinese Medicine Tablet Co., Ltd. (Shanghai, China) and identified by Dr. Guanglin Xu from Pharmacy Department, Shuguang Hospital Affiliated to Shanghai University of TCM. The preparation of SSR was performed as previously described [7, 8]. Briefly, the above nine commonly used Chinese herbs were mixed with distilled water in proportion and heated twice at 100°C for 1 h under continuous stirring condition. The aqueous extract was subjected to centrifugation at 1500 × *g*. The supernatant was merged and concentrated to prepare suspension containing 6 g/mL of the original drug by means of ethanol. The gavage dose (10 mL/kg, containing 6 g/mL of the original drug) in this study was determined on the basis of the previous studies [7, 8]. Losartan tablets (100 mg/tablet) were purchased from MSD Pharmaceutical Co., Ltd. (Hangzhou, China). Losartan was dissolved in saline and dosed by 6 mL/kg at the concentration of 5 mg/mL.

### 2.2. Rat 5/6 (A/I) Model and Animal Study Protocol

A rat CKD model with 5/6 (A/I) was created as previously described [7–10]. Four weeks after 5/6 (A/I) operation, 30 rats were randomized into three groups: 5/6 (A/I) group, 5/6 (A/I)+SSR group (treated with 10 mL/kg of SSR daily by gavage, *n* = 10), and 5/6 (A/I)+Losartan group (5/6 (A/I)+LOR, treated with 6 mL/kg of Losartan daily by gavage, *n* = 10). This study also included a group of 10 sham-operated rats. Eight weeks after treatment, rats were euthanized with sodium pentobarbital (40 mg/kg, i.p.). Kidney tissues were collected for histology and molecular analysis. The animal procedures were approved by the Animal Experiment Ethics Committee of Shanghai University of Traditional Chinese Medicine in accordance with the principles outlined in the NIH Guide for the Care and Use of Laboratory Animals.

### 2.3. Isolation of Cytosolic and Mitochondrial Fractions

Using a tissue mitochondria isolation kit (Inventbiotech, Beijing, China), the cytosolic and mitochondrial fractions were extracted at 4°C with a centrifugal column filter and by a multiple centrifugation method. About 30 mg of tissue was placed in a filter cartridge. The tissue was ground with a plastic rod for one minute by pushing the tissue against the surface of the filter repeatedly. The filter cartridge was centrifuged at 16,000 g for 30 seconds. The pellet was resuspended by vortexing briefly. The homogenates were first centrifuged at 700 g for one minute to remove cell debris and nuclei and then centrifuged at 16,000 g for 10 min to collect the supernatant as a cytosolic fraction. The pellet was resuspended in 200 *μ*L buffer B and then centrifuged at 8000 g for 5 min. The supernatant was further centrifuged at 16,000 g for 30 min to collect the pellet as a mitochondrial fraction.

### 2.4. Isolation of Cytosolic and Nuclear Fractions

Cytosolic and nuclear fractions were extracted at 4°C by the multiple centrifugation method, using a nuclear and cytoplasmic protein extraction kit (Beyotime, Shanghai, China). About 50 mg of tissue was cut into pieces and homogenized gently in the cytoplasmic protein extraction reagent with a glass tissue grinder. The homogenates were centrifuged at 1500 g for 5 min to collect the supernatant as a cytosolic fraction. The pellet was resuspended in 200 *μ*L cytoplasmic protein extraction reagent and then centrifuged at 16,000 g for 5 min to collect the supernatant as a cytosolic fraction. The pellet was resuspended in 50 *μ*L nuclear protein extraction reagent and then centrifuged at 16,000 g for 10 min to collect the supernatant as a nuclear fraction.

### 2.5. Caspase 3 Activity Analysis

The caspase 3 activity was detected with a caspase 3 activity assay kit (Jiancheng Bioengineering Institute, Nanjing, China). About 50 mg of tissue was ground in lysis buffer with a glass homogenizer and then centrifuged at 12,000 g for 15 min. The supernatant protein was collected and quantified by the Bradford method. The supernatant containing 200 *μ*g protein was incubated with 50 *μ*L reaction buffer and 5 *μ*L caspase 3 substrate at 37°C for 4 h. The caspase 3 activity was measured at absorbance of 405 nm.

### 2.6. Immunoprecipitation Analysis

Immunoprecipitation analysis was performed as previously described [7]. Briefly, kidney tissues were lysed on ice for 15 min in lysis buffer. Approximately 300 *μ*g of total protein was incubated overnight at 4°C with anti-P53 (Santa Cruz, USA) followed by precipitation with 70 *μ*L of protein A/G-Plus-Agarose (Santa Cruz, USA) for 4 h at 4°C. Nonspecific IgG (Proteintech) was used as the control. The precipitated complexes were washed in immunoprecipitation buffer and then resuspended in 30 *μ*L of 2× loading buffer and boiled for 5 min.

### 2.7. Renal Histology and Immunohistochemical (IHC) and Hoechst 33342 Staining

After embedding kidney tissues into paraffin, the 3 *μ*m sections were used for hematoxylin and eosin (HE) staining according to the standard protocol. Histopathological changes were scored as follows: 0, no damage; 1, <25%; 2, 25-50%; 3, 50-75%; and 4, >75%. IHC staining was performed as previously described [8]. The primary antibody for IHC staining was anti-*α*-SMA (1 : 100, Boster, China). 4 random fields per section were selected for the analysis of semiquantification by Image-Pro Plus 6.0 software.

The Hoechst 33342 staining (Beyotime, Shanghai, China) was used to identify the morphology of apoptotic nuclei. The sections were deparaffinized and then exposed to the Hoechst 33342 solution for 5 min at 37°C in the dark. The positive nuclei were observed with a fluorescence microscope (Nikon Eclipse80i, Japan) at 200x magnification.

### 2.8. Western Blot

An immunoblotting assay was performed as previously described [7, 8]. The primary antibodies used were anti-cytochrome C (1 : 1000, Abcam, UK), anti-Bcl2 (1 : 500, Genspan, USA), anti-Bax (1 : 1000, Abcam, UK), anti-SDHB (1 : 1000, Abcam, UK), anti-parp (1 : 1000, CST, USA), anti-Puma (1 : 500, Santa Cruz, USA), anti-ATPB (1 : 1000, Proteintech, USA), anti-caspase 9 (1 : 1000, Abcam, UK), anti-P53 (1 : 1000, CST, USA), anti-P-p53 (1 : 1000, Abcam, UK), and anti-Bcl-xL (1 : 1000, Abcam, UK). Lamin B1 (1 : 1000, ABclonal, China), VDAC1 (1 : 1000, Abcam, UK), and Gapdh (1 : 2000, Proteintech, USA) were used as the controls. The antigens on the blots were visualized by using the enhanced chemiluminescence kit (Thermo Fisher Scientific, USA).

### 2.9. Real-Time PCR

Total RNA was extracted from kidney tissues using the TRIzol reagent (Beyotime, Shanghai, China) and reverse transcribed to cDNA using a TaqMan RNA Reverse Transcription Kit (Takara, Dalian, China) according to the manufacturer's instructions. qPCR was performed on an ABI StepOnePlus Real-Time PCR System (Applied Biosystems, Foster City, USA) with SYBR Green Master Mix (Yeasen, Shanghai, China) according to the instructions provided with the kit. Relative expression was determined by the 2^−ΔΔCT^ method. *β*-Actin was used as an internal control. The PCR reaction conditions were as follows: 95°C for 5 min, followed by 40 cycles for 10 s at 95°C and 30 s at 60°C. The primers used were as follows: Puma, forward ACTGCCAGCCTTGCTTGTC and reverse AGTCCTTCAGCCCTCCCTTC; Bax, forward GGCGATGAACTGGACAAC and reverse CCGAAGTAGGAAAGGAGG; Noxa, forward GTTACCGCCTGAATTCGCAG and reverse AGTTATGTCCGGTGCACTCC; COX I, forward CACATGAGCAAAAGCCCACT and reverse ACGGCCGTAAGTGAGATGAA; and *β*-actin, forward GAGAGGGAAATCGTGCGT and reverse GGAGGAAGAGGATGCGG.

### 2.10. Statistical Analysis

All data were presented as mean ± SEM and analyzed by one-way analysis of variance with LSD-t multiple comparison, using SPSS software (version 18.0, SPSS Inc., Chicago, USA). *P* < 0.05 was considered statistically significant.

## 3. Results

### 3.1. SSR Attenuated Renal Injury and Fibrosis in the CKD Model

**Figure 1:**
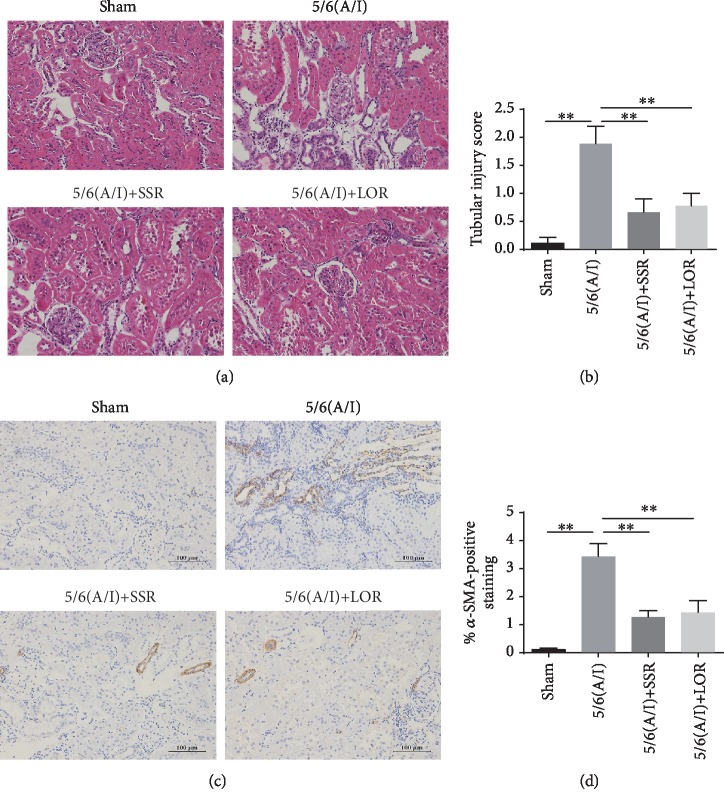
SSR attenuated renal injury and fibrosis in the CKD model. (a) Representative photomicrographs of hematoxylin and eosin (HE) staining. 200x magnification. (b) The severity of tubular damage was evaluated by the percentage of injured renal tubules (*n* = 4). (c) Representative photomicrographs of *α*-SMA expression detected by IHC. 200x magnification. (d) Quantitative analysis of *α*-SMA-positive staining (*n* = 3). Values are mean ± SEM. ^∗^*P* < 0.05, ^∗∗^*P* < 0.01.

Our previous studies have demonstrated that SSR markedly improved renal function and downregulated the expression of extracellular matrix (ECM) proteins [8]. In this study, we further estimated the severity of renal tubular injury and fibrosis. In histology, the 5/6 (A/I) group showed the extensive tubular injury, including the loss of brush border, tubular dilation and distortion, and inflammatory cell infiltration (Figure 1(a)). Quantitative analysis demonstrated that tubular damage in the 5/6 (A/I) group was significantly higher than that in the sham group (Figure 1(b)). However, SSR treatment significantly improved renal tubular injury. Furthermore, we performed IHC staining to evaluate the level of *α*-smooth muscle actin (*α*-SMA) protein, the marker of fibroblast activation. As shown in Figures 1(c) and 1(d), 5/6 (A/I) operation obviously increased the accumulation of *α*-SMA protein and SSR showed significant reduction in interstitial fibrosis when compared with the model group, which verified the renoprotective effects of SSR in kidney injury.

### 3.2. SSR Blocked Renal Apoptosis in the Rat CKD Model

**Figure 2:**
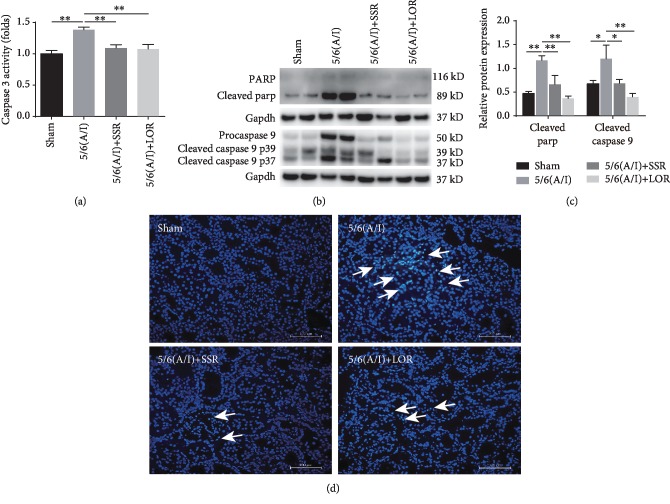
SSR blocked renal apoptosis in the rat CKD model. (a) The remnant kidney tissues were collected to detect the caspase 3 activity with an activity test kit (*n* = 4). (b) Representative Western blots demonstrating decreased cleaved Parp and caspase 9 after SSR treatment. (c) Quantification of cleaved Parp and cleaved caspase 9 levels (*n* = 4). (d) Representative microphotographs of apoptotic cells (marked by arrows) identified by Hoechst 33342 staining. 200x magnification. Values are mean ± SEM. ^∗^*P* < 0.05, ^∗∗^*P* < 0.01.

As a key executor of apoptosis, caspase 3 is implicated in the proteolysis of many critical proteins [11]. In this study, we first detected the level of caspase 3 activity. Compared with the sham group, rats receiving 5/6 (A/I) surgery showed markedly increased activity of caspase 3 (Figure 2(a)). SSR treatment for 8 weeks blocked 5/6 (A/I) injury-increased caspase 3 activity. As shown in Figures 2(b) and 2(c), SSR also inhibited the cleavage of parp, a specific substrate for caspase 3 and a biomarker of apoptosis [12]. The mitochondrial pathway of apoptosis requires activation of caspase 9, which then activates caspase 3 [13]. In this study, we found by immunoblotting analysis that SSR normalized cleaved caspase 9 content increased by 5/6 (A/I) injury (Figures 2(b) and 2(c)). Using the TUNEL assay, our previous studies reported that SSR treatment for 8 weeks dramatically reduced the number of apoptotic cells in the 5/6 (A/I) hypoxia model [8]. In the present study, we further analyzed the morphology of apoptotic nuclei by Hoechst 33342 staining. As shown in Figure 2(d), the normal nuclei were uniformly stained without nuclear condensation or fragmentation and the apoptotic cells showed the abnormal nuclear size and nuclear fragmentation or condensation. The morphology of nuclear abnormality induced by 5/6 (A/I) operation was obviously ameliorated by SSR treatment.

### 3.3. SSR Inhibited the Mitochondrial Accumulation of Proapoptotic Bax and Puma Proteins in the CKD Model

**Figure 3:**
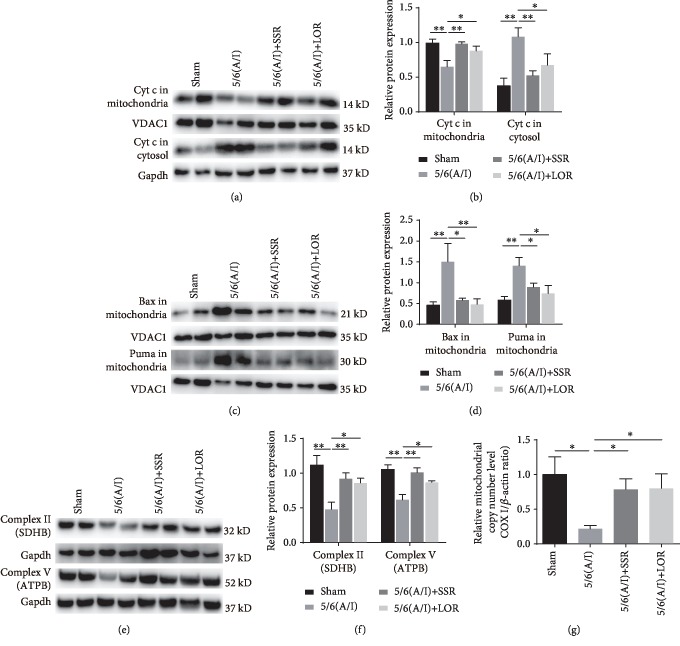
SSR inhibited the mitochondrial accumulation of proapoptotic Bax and Puma proteins in the CKD model. (a) Representative immunoblot demonstrating reduced translocation of cytochrome c (Cyt c) 8 weeks after SSR treatment in 5/6 (A/I) rats. Gapdh and VDAC1 were used as loading controls. (b) Quantification of the relative protein levels of Cyt c in the mitochondria and cytosol (*n* = 4). (c) Representative immunoblot showing mitochondrial Bax and Puma protein content. (d) Quantification of mitochondrial Bax and Puma levels (*n* = 4). (e) The protein levels of complex II (SDHB) and complex V (ATPB) subunits were determined by immunoblotting. (f) Quantification of SDHB and ATPB levels (*n* = 4). (g) The level of the mitochondrial copy number was determined by the ratio of the cytochrome c oxidase subunit I (COX I) gene to *β*-actin (*n* = 4). Values are mean ± SEM. ^∗^*P* < 0.05, ^∗∗^*P* < 0.01.

The mitochondrial pathway of apoptosis is mainly triggered by Bax accumulation in the mitochondria and subsequent release of apoptogenic factors, such as cytochrome c, from the mitochondrial intermembrane space [14]. We enriched the mitochondrial fraction from the remnant kidneys and analyzed the translocation of Bax and cytochrome c proteins by immunoblotting. In the sham group, cytochrome c was mainly located in the mitochondria and the 5/6 (A/I) injury significantly increased the translocation of cytochrome c to the cytosolic fraction (Figures 3(a) and 3(b)). Compared with the sham group, rats in the 5/6 (A/I) group displayed a higher accumulation of Bax in the mitochondria (Figures 3(c) and 3(d)). Conversely, SSR treatment for 8 weeks markedly inhibited the accumulation of Bax in the mitochondria and the release of cytochrome c into the cytosol. In addition, we detected the level of p53 upregulated modulator of apoptosis (Puma), a proapoptotic BH3-only protein that was shown to localize to mitochondria and activate Bax [14], in the mitochondrial and cytosolic samples. Our results demonstrated that the accumulation of Puma in mitochondria showed the same pattern as that of Bax in each group (Figures 3(c) and 3(d)).

Furthermore, mitochondrial dysfunction is a well-known pathogenic factor in ischemic renal injury and the destruction of mitochondrial integrity is more sensitive to the insertion of Bax [15, 16]. Therefore, we detected the levels of mitochondrial respiratory chain-associated proteins and the mitochondrial copy number as determined by the ratio of the cytochrome c oxidase subunit І (COX І) gene to *β*-actin to estimate mitochondrial function in the remnant kidneys. Compared with the sham group, the SDHB and ATPB, the subunits of complex II and complex V [17], and mitochondrial copy number were significantly downregulated in the 5/6 renal (A/I) model group, which was dramatically reversed by SSR (Figures 3(e), 3(f), and 3(g)).

### 3.4. SSR Suppressed the Nuclear Translocation and Transactivation of p53 in the CKD Model

**Figure 4:**
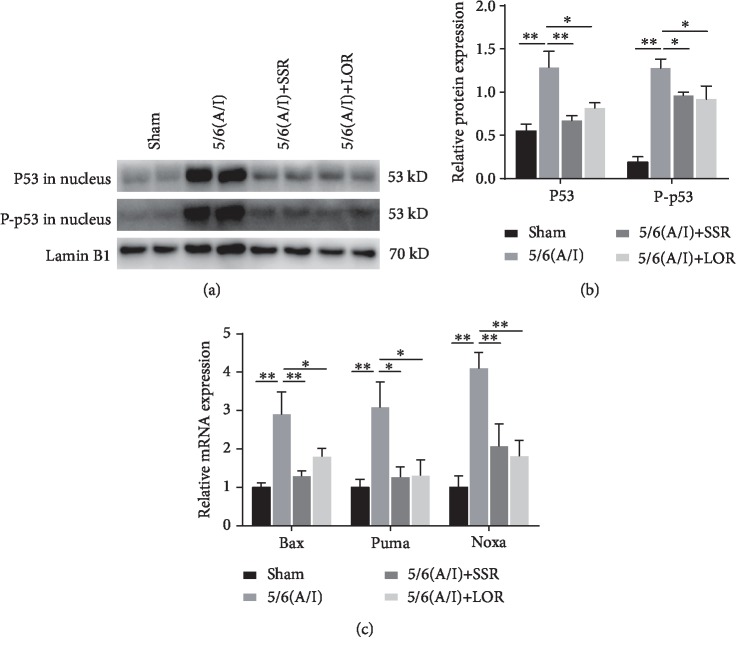
SSR suppressed the nuclear translocation and transactivation of p53 in the CKD model. (a) Representative immunoblot demonstrating decreased nuclear P53 protein content and phosphorylation (Ser15) 8 weeks after SSR treatment in 5/6 (A/I) rats. (b) Quantification of nuclear P53 protein content and phosphorylation normalized to lamin B1 protein (*n* = 4). (c) Normalized content of Bax, Puma, and Noxa mRNA 8 weeks after SSR treatment. The abundance of each mRNA was normalized to *β*-actin, and data were expressed as a fold increase over control (*n* = 4). Values are mean ± SEM. ^∗^*P* < 0.05, ^∗∗^*P* < 0.01.

Since p53 is the most critical molecule upstream of Puma and Bax and that the nuclear translocation of p53 represents its activation [18], we extracted the nuclei from the remnant kidney tissues to analyze the protein level of p53 in the nuclear fraction. We found that nuclear p53 content and its phosphorylation (Ser15) were significantly upregulated in rats after 5/6 (A/I) injury (Figures 4(a) and 4(b)). Furthermore, qRT-PCR results demonstrated that the mRNA expression of Puma, Bax, and Noxa, the direct targets of p53, was markedly upregulated in the 5/6 (A/I) model (Figure 4(c)). Importantly, SSR significantly inhibited the translocation of p53 from the cytosol to the nucleus and the expression of p53 target genes in 5/6 (A/I) rats.

### 3.5. SSR Inhibited the Mitochondrial Translocation and Activation of p53 in the CKD Model

**Figure 5:**
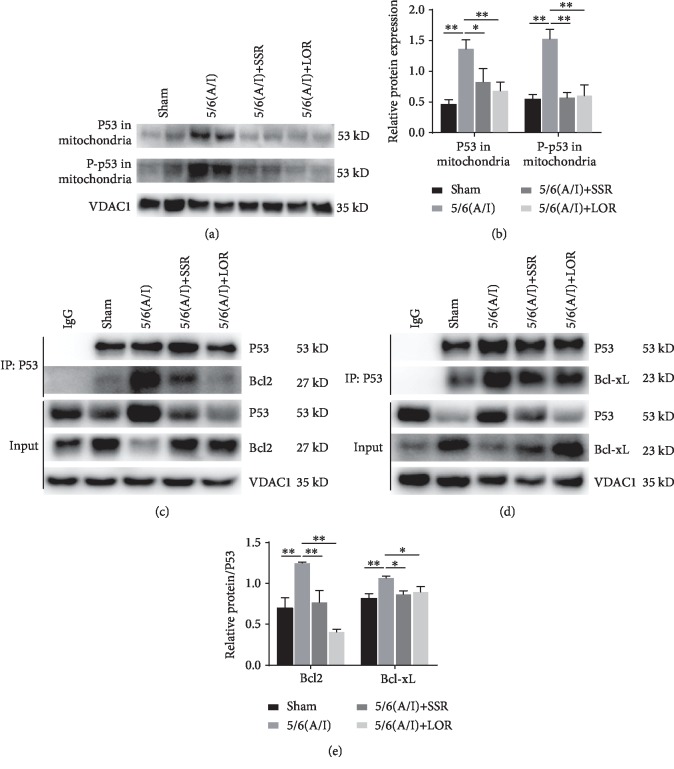
SSR inhibited the mitochondrial translocation and activation of p53 in the CKD model. (a) Representative immunoblot demonstrating p53 mitochondrial translocation. (b) Quantification of protein content of p53 and P-p53 (Ser15) in the mitochondria (*n* = 4). (c, d) Coimmunoprecipitation/Western blot demonstrating the interaction of p53 with Bcl-xL and Bcl-2 proteins in each group. (e) The ratio of Bcl-xL and Bcl-2 to precipitated P53 protein was calculated (*n* = 3). Values are mean ± SEM. ^∗^*P* < 0.05, ^∗∗^*P* < 0.01.

In addition to its transcriptional-dependent proapoptotic effect, p53 could also translocate to mitochondria and interact with Bcl-xL and Bcl-2 to inhibit their antiapoptotic effects [6, 19–21]. In this study, we found that the 5/6 (A/I) injury increased p53 and its phosphorylation (Ser15) content in the mitochondria, which was inhibited by SSR treatment for 8 weeks (Figures 5(a) and 5(b)). To assess the interaction of p53 with antiapoptotic Bcl-xL and Bcl-2 proteins, p53 was immunoprecipitated and the degree of interaction was analyzed by immunoblotting. As shown in Figures 5(c) and 5(d), coimmunoprecipitation/immunoblotting studies demonstrated that p53 directly interacted with Bcl-xL and Bcl-2 in the mitochondria. Quantitative analysis showed that the binding of p53-Bcl2 or p53-Bcl-xL was increased after 5/6 (A/I) injury and SSR significantly inhibited the interaction of p53 with Bcl-xL and Bcl-2 (Figure 5(e)).

## 4. Discussion

In this study, we demonstrated that SSR blocked apoptosis induced by 5/6 (A/I) injury by inhibiting the accumulation of Bax and Puma in the mitochondria. As a result, release of cytochrome c into the cytosol and caspase 3 activity were decreased. In addition, SSR inhibited the nuclear translocation and transcriptional activity of p53 in 5/6 (A/I)-injured rats, which was shown by the decreased expression of Puma, Bax, and Noxa mRNA. Importantly, coimmunoprecipitation/immunoblotting analysis demonstrated that SSR not only significantly reduced the translocation of P53 to mitochondria but also inhibited the direct interaction of P53 with antiapoptotic Bcl-2 and Bcl-xL proteins.

Previous studies demonstrated that in different disease models, inhibition of apoptosis showed significant renal protective effects. Du et al. [22] reported that the activation of vitamin D receptor protected against lipopolysaccharide- (LPS-) induced acute kidney injury by blocking renal tubular epithelial cell apoptosis. Huang et al. [23] demonstrated that L3MBTL2, a novel polycomb group protein, improved renal injury by the p53 apoptosis pathway in renal tubular cells. Peng et al. [16] reported that p53 and the mitochondrial pathway of apoptosis were involved in the susceptibility of diabetic models to ischemic acute kidney injury. In addition, many herbs and active ingredients in SSR have shown antifibrotic effects in different CKD models. Cai et al. [24] reported that Salvia miltiorrhiza significantly improved renal function and interstitial fibrosis in an adenine-induced CKD model. Chen et al. [25] demonstrated that icariin, an active polyphenol of the Epimedium genus, protected against CKD-associated renal fibrosis in a unilateral ureteral obstruction model. Li et al. [26] reported that a polysaccharide from the stem of Codonopsis pilosula attenuated renal injury in an ischemia/reperfusion injury model by inhibiting the proinflammatory cytokine TNF-*α* release. Rheum and its extracts have nephroprotective effects in the CKD model induced by nephrotoxicity and adenine [27, 28]. These studies prompted us to further study the possible role of apoptosis in the 5/6 (A/I) injury model, as well as the mechanisms of SSR attenuating renal injury and fibrosis. The mitochondria, as the central organelle of the intrinsic apoptotic pathway, are regulated by pro- and antiapoptotic Bcl-2 family members [29]. Upon stress, the integrity of the outer mitochondrial membrane (OMM) is impaired, which is sensitized to the accumulation and insertion of Bax and subsequently leads to the release of cytochrome c into the cytosol [30, 31]. Our previous studies showed that 5/6 (A/I) injury increased the accumulation of Bax in the mitochondria and release of cytochrome c into the cytosol [10]. In this study, we showed that SSR markedly inhibited the intrinsic apoptotic pathway, improved the pathological morphology of the nucleus, and lowered caspase 3 activation, which could be associated with renal protective effects of SSR. In addition, as the main oxygen consumption organelle, the mitochondria are uniquely dependent on the continuous availability of oxygen for the function of the electron transport chains [32]. During hypoxia, the dysfunction and dynamic disruption of mitochondria attract Bax attack and insertion and ensuing apoptosis [33]. Therefore, we detected the mitochondrial copy number, a biomarker for mitochondrial dysfunction, and the levels of mitochondrial electron transport chain proteins. SSR significantly increased the mitochondrial copy number and protein levels of complex II and complex V subunits, which could be helpful to maintain mitochondrial integrity and resist Bax insertion and cytochrome c release.

Previous studies have reported that persistent hypoxia could be the main trigger of p53 activation in the process of renal injury and p53-mediated induction of Puma and Bax was responsible for apoptosis in ischemic kidneys [34–37]. However, it is important that, in the process of the mitochondrial apoptosis pathway, the suborganellar localization of p53 affects its function. In the nucleus, transcriptional activation of p53 upregulates the expression of Puma and Bax involved in the mitochondrial apoptosis pathway [38]. Therefore, we isolated the nuclear and cytoplasmic components from the remnant kidney tissues to detect the translocation of p53 to the nucleus. Our results showed that the levels of p53 and its phosphorylation in the nuclear fraction were dramatically elevated in the 5/6 (A/I) model. SSR significantly suppressed the nuclear translocation and transactivation of p53, evidenced by the downregulation of Bax, Puma, and Noxa mRNA expression. More importantly, in response to stress, cytoplasmic p53 is directly involved in the mitochondrial pathway of apoptosis by translocation to mitochondria. P53 in mitochondria can directly bind to the antiapoptotic members Bcl-xL and Bcl-2 and neutralize their inhibitory effects on proapoptotic Bax to stimulate mitochondrial outer membrane permeabilization (MOMP) and subsequent apoptosis. In this study, we found that SSR inhibited the accumulation and phosphorylation of p53 in the mitochondria. Furthermore, using the coimmunoprecipitation analysis, we showed that 5/6 (A/I) operation increased the binding of p53 to Bcl2 or Bcl-xL in the mitochondria, which was reversed by SSR.

In conclusion, SSR could attenuate apoptosis in the rat CKD model by the mitochondrial pathway of apoptosis. Furthermore, activation of p53-induced target genes and transcriptional-independent apoptotic activity of p53 could amplify the intrinsic pathway of apoptosis, which was inhibited by SSR treatment for 8 weeks. However, the relationship between p53 transcriptional-dependent and transcriptional-independent proapoptotic function and the regulatory effects of SSR on them need to be further clarified in the future study.

## Data Availability

The data used to support the findings of this study are available from the corresponding author upon request.
